# Epicardial Adipocytes in Cardiac Pathology and Healing

**DOI:** 10.1007/s10557-024-07637-2

**Published:** 2024-11-06

**Authors:** Vy La, Vishnu Nair, Sini Sunny, Peyman Benharash, Finosh G. Thankam

**Affiliations:** 1 Department of Translational Research, College of Osteopathic Medicine of the Pacific, Western University of Health Sciences, 309 E. Second Street, Pomona, CA 91766-1854 USA; 2 Department of Molecular, Cell, & Developmental Biology, University of California, Los Angeles, CA 90095 USA; 3 Department of Pathology, University of Alabama at Birmingham, Birmingham, AL 35294 USA; 4 Division of Cardiac Surgery, David Geffen School of Medicine, University of California, Los Angeles, CA 90095 USA

**Keywords:** Epicardial adipose tissue, Coronary artery disease, Stem cells, Cardiac regeneration

## Abstract

Implications of epicardial adipose tissue (EAT) on the development of coronary artery disease (CAD) have garnered recent attention. Located between the myocardium and visceral pericardium, EAT possesses unique morphological and physiological contiguity to the heart. The transcriptome and secretome of EAT differ from that of other fat stores in the body. Physiologically, EAT protects the adjacent myocardium through its brown-fat-like thermogenic function and rapid fatty acid oxidation. However, EAT releases pro-inflammatory mediators acting on the myocardium and coronary vessels, thus contributing to the development and progression of cardiovascular diseases (CVD). Furthermore, EAT-derived mesenchymal stromal cells indicate promising regenerative capabilities that offer novel opportunities in cell-based cardiac regeneration. This review aims to provide a comprehensive understanding and unraveling of EAT mechanisms implicated in regulating cardiac function and regeneration under pathological conditions. A holistic understanding of the multifaceted nature of EAT is essential to the future development of pharmacological and therapeutic interventions for the management of CVD.

## Introduction

Cardiovascular diseases (CVD) are the leading cause of morbidity and mortality worldwide [1]. Excess adiposity poses a significant risk to developing CVD, impacts endothelial homeostasis, increases insulin resistance, and triggers systemic inflammation [2]. Current research on cardiometabolic diseases has shifted from an obesity-centered focus to CVD prevention using cardiac-specific adiposity. Adipose tissue surrounding the heart is classified as epicardial adipose tissue (EAT), pericardial, paracardial, and perivascular. Epicardial fat is located below the visceral pericardium; pericardial fat is in between the visceral and parietal pericardial layers; paracardial fat is outside the parietal pericardium; and perivascular fat is around the coronary arteries, irrespective of location [3]. The role of EAT in obesity and CVD has rapidly grown, with this tissue now being recognized as metabolically active and exerting modulatory effects in CVD [4].

EAT is present on 80% of the heart surface and directly in contact with major coronary arteries and their branches. Also, EAT is most abundant in the atrioventricular (AV) grooves, interventricular grooves, and the RV lateral wall [5]. Mechanically, EAT acts as an elastic cushion that protects the coronary arteries against excessive distortion caused by arterial pulse and myocardial contraction [6]. EAT upregulates the brown adipose tissue thermogenic marker, uncoupling protein 1 (UCP1) more than the other fat depots. This permits heat production to protect the myocardium and coronary artery from hypothermia damage [7]. EAT is twice as metabolically active as other fat depots, given its proximity to the demanding heart, with higher levels of fatty acid uptake and release due to lipolysis [8]. Fascial layers are absent between EAT and the underlying myocardium, indicating unobstructed microcirculation between the two, facilitating paracrine and vasocrine communication [9]. In healthy conditions, EAT releases cytokines to nourish the myocardium, while in pathological conditions, EAT releases pro-inflammatory mediators that target the myocardium and coronary vessels, contributing to the development and progression of CVD [10].

EAT dysfunction/disorders represent a modifiable risk factor and potential therapeutic target for drugs with cardiovascular benefits. Glacobellis found that when glucagon-like peptide-1 (GLP-1) and sodium-glucose cotransporter 2 (SGLT2) reduced left atrial and coronary EAT thickness, EAT inflammation was decreased with a concomitant increase in fatty acid oxidation, preventing atrial fibrillation and coronary artery disease, especially in patients with high BMI [11]. Similarly, Packer et al. demonstrated that in patients at risk of heart failure with preserved ejection fraction, certain drugs (e.g., statins, mineralocorticoid antagonists, sodium-glucose co-transporter 2 inhibitors) that improved proinflammatory characteristics of epicardial fat, significantly reduced the development of heart failure [10]. These observations suggest the critical role of EAT as an important target for cardiovascular metabolic disorders. This review focuses on a comprehensive overview of the role of EAT as a quantifiable risk indicator in the development and progression of CVD. By exploring the intricate interplay of EAT and its neighboring structures, such as the coronary arteries and myocardium, this review unravels the prospective mechanisms by which EAT impacts cardiac function under pathological conditions.

### Adipose Tissue Biology

**Fig. 1:**
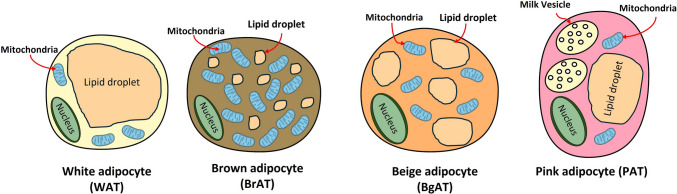
Types of adipose tissue: White adipocytes are characterized by their large lipid droplet and sparse mitochondria. Brown adipocytes are rich in mitochondria, with numerous, smaller lipid droplets. Beige adipocytes form a common ground between the fat types, containing medium sized lipid droplets and frequent mitochondrial presence. Pink adipocytes, indicated by their milk vesicles, are prominent in mammary glands during pregnancy and lactation

Adipose tissue is classified based on its visually distinct color profile, cell structure, and specialized functions into four types: white adipose tissue (WAT), brown adipose tissue (BrAT), beige adipose tissue (BgAT), and pink adipose tissue (PAT) [8] (Fig. 1, Table 1). WAT is the most abundant type of adipose tissue, constituting subcutaneous fat, visceral fat, and bone marrow fat in humans. WAT is spherical and comprises a single large lipid droplet with minimal mitochondria in its periphery. WAT serves as an energy reservoir, primarily in the form of triglycerides. Hormones, including insulin and glucagon, tune WAT to undergo adipogenesis or adipolysis, respectively, based on energy demand. Also, WAT secretes several biologically active factors known as adipokines that regulate the energy balance, nutrient satiety, inflammatory response, and steroid hormone metabolism. Furthermore, WAT acts as a cushion, protecting body parts by dissipating force and insulating the body during extreme cold exposure. WAT is characterized by markers including Rip140, resistin, leptin, and hocx9 [12].

**Table 1:** Properties and biomarkers of adipose tissue

	White adipose tissue (WAT)	Brown adipose tissue (BrAT)	Beige adipocytes (BgAT)	Pink adipocytes (PAT)
Main function	Energy storage	Non-shivering heat generation, thermogenesis	Shivering heat generation	Milk production and secretion
Location	Subcutaneous, visceral	Upper back, neck, above clavicles	Thoracic region, subcutaneous	Subcutaneous, breast tissue
Primary markers	ADIPOQ AQP7 HOCX9 Leptin RIP1140 Resistin	CIDEA UCP1 PGC-1 PRDM16 CD137 TMEM26	AQP7 CITED1 FGF21 HOCX9	ADIPOQ Leptin Prolactin

BrAT presents mostly during fetal development, infants, postnatally, and hibernation [13]. BAT accounts for < 1% of body weight and is centrally located in the upper back, above the clavicles, around the vertebrae, and in the mediastinum. BrAT preserves an ellipsoidal shape that contains several small lipid droplets and a large density of mitochondria, giving the cell a brownish hue. Compared to WAT, BrAT has more blood vessels and more abundant cytochrome stores in the cytoplasm. In contrast to WAT, which stores energy, BrAT generates energy via mitochondria-directed oxidative phosphorylation. BrAT augments uncoupling protein 1 (UCP-1) levels, an ATP-generating protein gradient devoted to adaptive thermogenesis for non-shivering heat production, critical for body temperature maintenance [14]. In adults, non-shivering thermogenesis is secondary to shivering thermogenesis and is achieved by the contraction of skeletal muscles. Other major markers of BrAT include PRDM16, PGC-1, CIDEA, and ADIPOQ [15].

BgAT resides within WAT, possessing both WAT and BrAT-derived characteristics. In contrast to BrAT, BgAT generates shivering-heat production, known as cold-induced thermogenesis. The loss of beige adipogenesis with increased age has been associated with the loss of energy-expanding capacity and the obesity-prone phenotype in older populations. BgAT and WAT marker overlap, such as Hoxc8, Hoxc9, Aqp5, Aqp7, and Aqp9, is expected, with near absence from brown fat [15], 16. Other markers unique to BgAT include FGF21 and CITED1.

PAT develops exclusively in females and mainly exists in the breast. PAT is expected to be transdifferentiated from WAT via upregulation of secreted phosphoprotein 1 (SPP1) during pregnancy, lactation, and the post-lactation period to promote milk production and secretion. PAT is specialized in the mammary gland alveolar epithelial cells with well-developed secretory structures, including the Golgi apparatus and the accumulated endoplasmic reticulum, giving them their pink hue [17]. The presence of PAT is confirmed by the whey acidic protein (WAP) gene, a marker of the milk-producing epithelial mammary gland. The transition from WAT to PAT is confirmed by the decreased expression of the Plin1 and 2 genes as pregnancy progresses [18]. The known prominent markers for PAT are ADIPOQ, leptin, and prolactin.

### Adipogenesis and Differentiation

#### Differentiation of WAT from Progenitor Cells

WAT predominantly arises from the Myf5-negative lineage (paraxial mesoderm) [19]. WAT is derived from the adipogenic lineage, whereas BrAT is derived from the myogenic lineage. Although the adipocytes originate from different lineages, the process of adipogenic differentiation involves common transcriptional cascades that involve PPAR-γ and C/EBPs [20]. The binding of C/EBP-β to DNA leads to increased levels of C/EBP-α and PPAR-γ, acting together as transcriptional activators. Upon expression, C/EBP-α and PPAR-γ positive feedback on each other is a critical step in acquiring the mature adipocyte phenotype. Moreover, PPAR-γ regulates transcription, inducing and maintaining the differentiated state of adipocytes (lipid metabolism, glucose metabolism, and insulin sensitivity) [21]. The dominant negative form of PPAR-γ leads to de-differentiation and the loss of lipid accumulation in differentiated adipocyte cells [22]. Sanchez-Gurmaches et al. demonstrated that white adipocytes are derived from the Myf5-positive lineage mesenchymal progenitors [23]. The relative contribution of Myf5-positive lineage cells to WAT varies among different WAT depots. Additionally, Pax3 (an upstream regulator of Myf5 during myogenesis) lineage cells contribute to a subset of WAT in different depots. Owing to the Myf5-lineage origin of brown adipocytes, it is logical that the Myf5-lineage progenitors are more likely to give rise to the adaptive BgAT [24].

#### Differentiation into Brat from Progenitor Cells

In contrast to white adipocytes, brown adipocytes share a progenitor cell, myogenic factor 5 (Myf5-positive, lateral mesoderm), with skeletal muscle. The differentiation process of brown preadipocytes into BrAT is regulated by transforming growth factor-β family proteins such as bone morphogenetic protein (BMP)-7 and myostatin [25]. Wnt signaling suppresses the differentiation of the preadipocytes into brown adipocytes [26]. C/EBP-β and PR domain containing 16 (PRDM16) transcriptional factors play a vital role in differentiating BrAT. In the Myf5+ myogenic lineage, the PRDM16 and C/EBP-β transcriptional complex induces the expression of PPAR-γ and PPAR-γ coactivator 1 alpha (PGC-1α), which subsequently induces the differentiation of BrAT [27].

#### Formation of Beige/Brite Adipocyte (“Browning”)

**Fig. 2:**
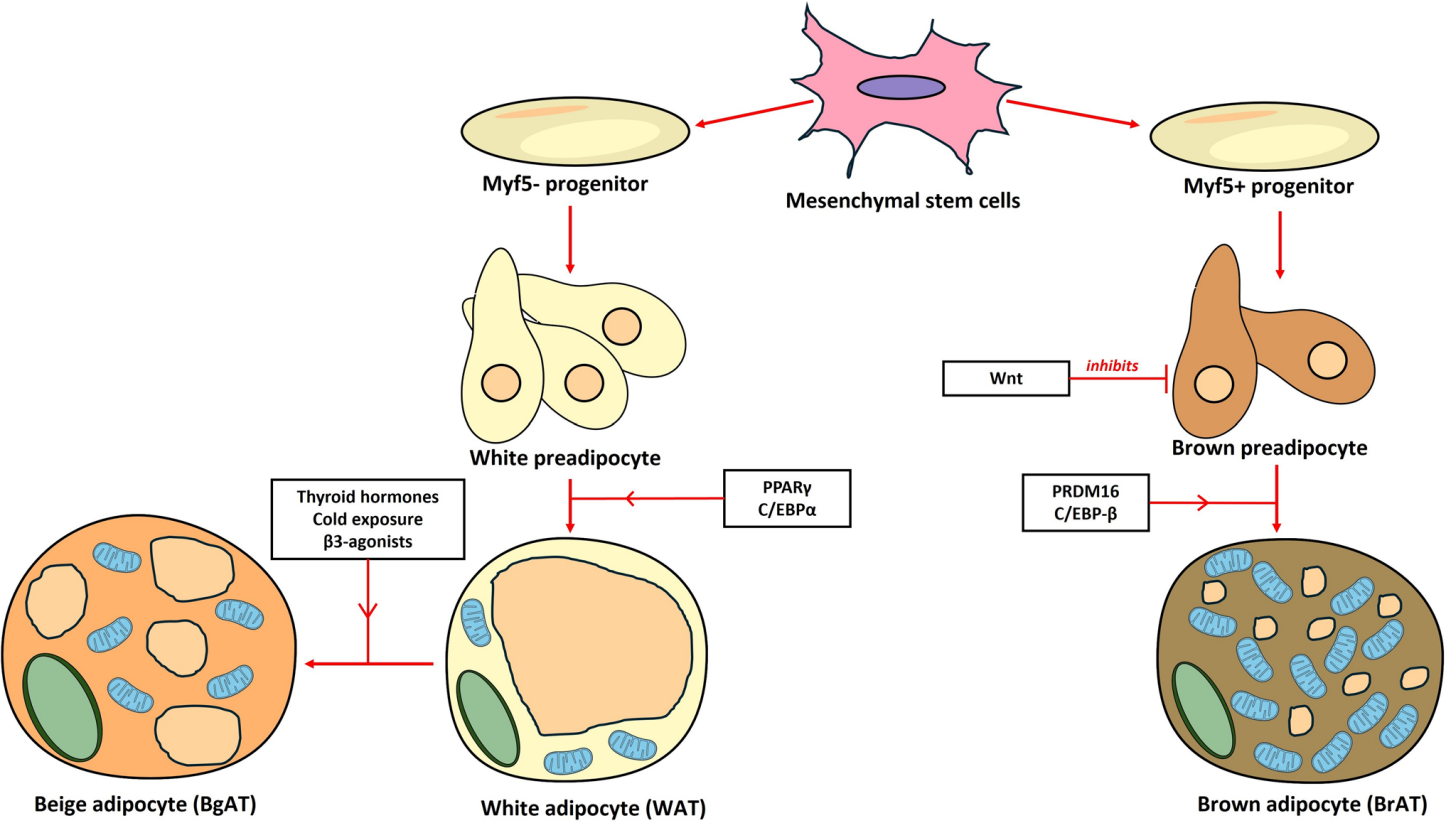
Adipocyte differentiation: Mesenchymal stem cells differentiate into Myf5− and Myf5+ progenitors, which develop into white and brown preadipocytes, respectively. White preadipocytes can further develop into mature white adipocytes, and if exposed to thyroid hormones, cold, or β3-agonists, beige adipocytes. Further brown preadipocyte development is inhibited by Wnt, however, when subjected to PRDM 16 and C/EBP-β, mature brown adipocytes form

WAT depots switch between energy storage and expenditure via a process called “browning.” Thus, these depots shift from a WAT phenotype to a BAT-like phenotype regarding features such as morphology, gene expression pattern, and mitochondrial respiratory activity under some specific stimuli. Under basal conditions, these beige/brite cells usually exhibit characteristics closer to the WAT phenotype, including large lipid droplets and the lack of UCP1 expression. However, in response to certain stimuli (chronic cold exposure or β3-adrenergic activators such as norepinephrine), beige/brite cells transform into cells having BAT-like characteristics, such as multilocular/small lipid droplets and UCP1 expression [28]. Thyroid hormones induce WAT browning [29]. Specifically, triiodothyronine (T3) and triiodothyracetic acid (TRIAC) regulate the WAT browning process and induce ectopic expression of UCP1 in abdominal WAT depots [30]. Since classical BAT has a fixed mechanism to control energy homeostasis, beige/brite cells offer a more flexible way to regulate body temperature and energy balance. The differentiation pattern of adipose tissues is depicted in Fig. 2.

### Adipose Tissues in Metabolic and Cardiac Diseases

Dysregulation of adipose tissue results in an imbalance between caloric intake and energy expenditure as existing adipocytes in the tissue fail to capture and retain circulating lipids. Adipose hyperplasia and hypertrophy occur as an adaptive mechanism, leading to proliferative adipose tissue expansion, the hallmark of obesity. Adipose tissue distribution was previously found to be a strong predictor of the development of metabolic syndrome [8] [31], 32, [33]. Since visceral fat is metabolically active and is constantly releasing free fatty acids into the portal circulation, obese patients with predominantly visceral (aka central) adipose tissue expansion have an increased risk of developing cardiovascular and metabolic diseases [34].

Hypoxia triggers a particular cellular stress response, including phenotype switching in adipocytes from anti-atherogenic to pro-inflammatory. Pro-atherogenic adipose tissue releases inflammatory adipokines (TNFα, IL-1β, IL-6, monocyte chemoattractant protein 1) and infiltration of immune cells. The eventual development of a chronic local or systemic low-grade inflammation with these cytokines results in lipid spillover and glucotoxicity. These pathologies contribute to insulin resistance, a critical factor in the pathogenesis of type 2 diabetes (T2DM) and CVD [35]. Furthermore, lipid spill-over infiltrates systemic circulation, leading to tissue damage, most importantly, the heart.

Chronic inflammatory conditions such as obesity and T2DM are associated with fat accumulation in the heart, rendering ectopic fat in the heart a strong predictor of CVD. Cardiac tissue utilizes FFA for metabolism; however, excess myocardial fatty acid oxidation leads to lipotoxic product accumulation. These products directly promote changes in cardiac function by affecting myocardial vasculature and indirectly through obesity-related comorbidities [36], 37. Obesity directly affects myocardial fat accumulation and fibrosis that develop left ventricular diastolic function and heart failure with preserved ejection fraction (HFpEF) [38]. Comorbidities associated with obesity, such as diabetes, sleep apnea, and hypoventilation syndrome, exacerbate the risk for pulmonary hypertension, atrial fibrillation, right ventricular and LV failure, and sudden cardiac death [36].

### Adipokines-Derived Signaling

Adipocyte communication is mediated by the secretion of adipocytokines and microvesicles, that engage in different complex signaling pathways of differentiation, metabolism, inflammation, and systemic homeostasis. Several transcription factors regulate adipogenesis by stimulating the differentiation of mesenchymal stromal cells (MSCs) and preadipocytes to produce mature adipocytes. MSCs generate adipoblasts that differentiate into preadipocytes under the influence of multiple transcription factors such as preadipocyte factor-1 (Pref-1), sterol regulatory element-binding protein 1 (SREBP-1), and peroxisome proliferator-activated receptor gamma (PPARγ). Preadipocytes differentiate into immature adipocytes and later mature adipocytes under the influence of CCAAT/enhancer-binding protein alpha (C/EBPα), adipocyte protein 2 (aP2), leptin, lipoprotein lipase (LPL), leukocyte differentiation antigen (CD36), and glucose transporter number 4 (GLUT4) [39].

Pro-inflammatory adipokines such as TNFα and several interleukins, notably IL-1β and IL-6, are elevated in adipose tissue inflammation and complications associated with obesity [40]. Other adipokines, such as leptin, adiponectin, and resistin, affect insulin function and the metabolism of lipids and glucose. Leptin acts as a satiety signal in the hypothalamus and regulates energy expenditure, which is crucial for controlling body weight. Resistin levels are elevated in obesity with the cellular glucose uptake inhibition, ultimately resulting in increased triglycerides and cholesterol levels in macrophages. Adiponectin exerts anti-oxidative, anti-inflammatory, and insulin-sensitizing properties, essential for glucose and lipid metabolism and the prevention of vascular remodeling. Notably, adiponectin enhances insulin activity, inhibits the production of pro-inflammatory adipokines (TNFα and IL-6), decreases lipid accumulation in the liver, and improves endothelial function via its anti-atherogenic properties [41].

Adipose tissue serves as a primary target for insulin action. Insulin promotes glucose uptake and fatty acid storage in adipocytes. During excessive cellular energy intake, subcutaneous adipose tissue becomes hypertrophic, leading to tissue malfunction and fibrosis. Some mediators of lipid formation, including protein kinase C (PKC) and ceramides, are activated by lipid overload, enhancing lipid accumulation in the liver and muscles, giving rise to insulin resistance [42]. Also, adipocytes display nuclear receptors important in the regulation of lipid and glucose metabolism known as peroxisome proliferator-activated receptors (PPARs).

Many recent studies consider extracellular vesicles (EVs) released by adipose tissue as an alternative pathway to maintain metabolic homeostasis and drive disease under certain pathological conditions [43–45]. For example, the cargo composition in WAT-derived EVs changes based on the nutritional status [44]. This is seen as EVs from fasted mice contain more electron transport chain proteins and fewer mitochondrial fatty acid oxidation proteins [46]. BrAT-secreted EVs regulate the function of other tissues, including the liver, through the transport of miRNA. However, there is a lack of knowledge regarding the composition of EVs and their role in BrAT. Selective sorting of EV cargo has significant implications for inter-tissue communication and disease progression, unlocking new avenues in obesity and metabolic disease treatment [46].

### EAT in Cardiac Pathology

EAT evolves from BrAT, providing a direct source of heat to the myocardium against unfavorable hemodynamic conditions such as hypothermia, ischemia, or hypoxia. During high energy demand, EAT transports FFA from epicardial fat into the myocardium via adipocyte fatty acid-binding protein (FABP4) to provide energy and protect the heart against high fatty acid levels [11]. Iacobellis et al. suggested that SGLT2 inhibitors and GLP1R agonists enhance FFA oxidation in the myocardium. In contrast, GLP1 agonists further induce brown fat differentiation and pre-adipocyte differentiation, improving myocardial insulin sensitivity, suggesting the potential for novel pharmaceutical modulation in reducing EAT inflammation and improving cardiometabolic outcomes [11]. Varying patient responses to drugs including SGL2 inhibitors and GLP1R agonists demand pharmacogenomic testing to personalize treatment modality by identifying specific genetic variants influencing the drug metabolism. It is likely that the potential redistribution of fats occurs following the treatment of EAT with such drugs. Also, the subcutaneous and ectopic fat accumulation in the liver and muscle, interfering with metabolic processes, and thermogenesis are expected [47]. Hence, proper dosage and toxicology of EAT targeting medication warrants intense monitoring to ensure physiological safety.

**Fig. 3:**
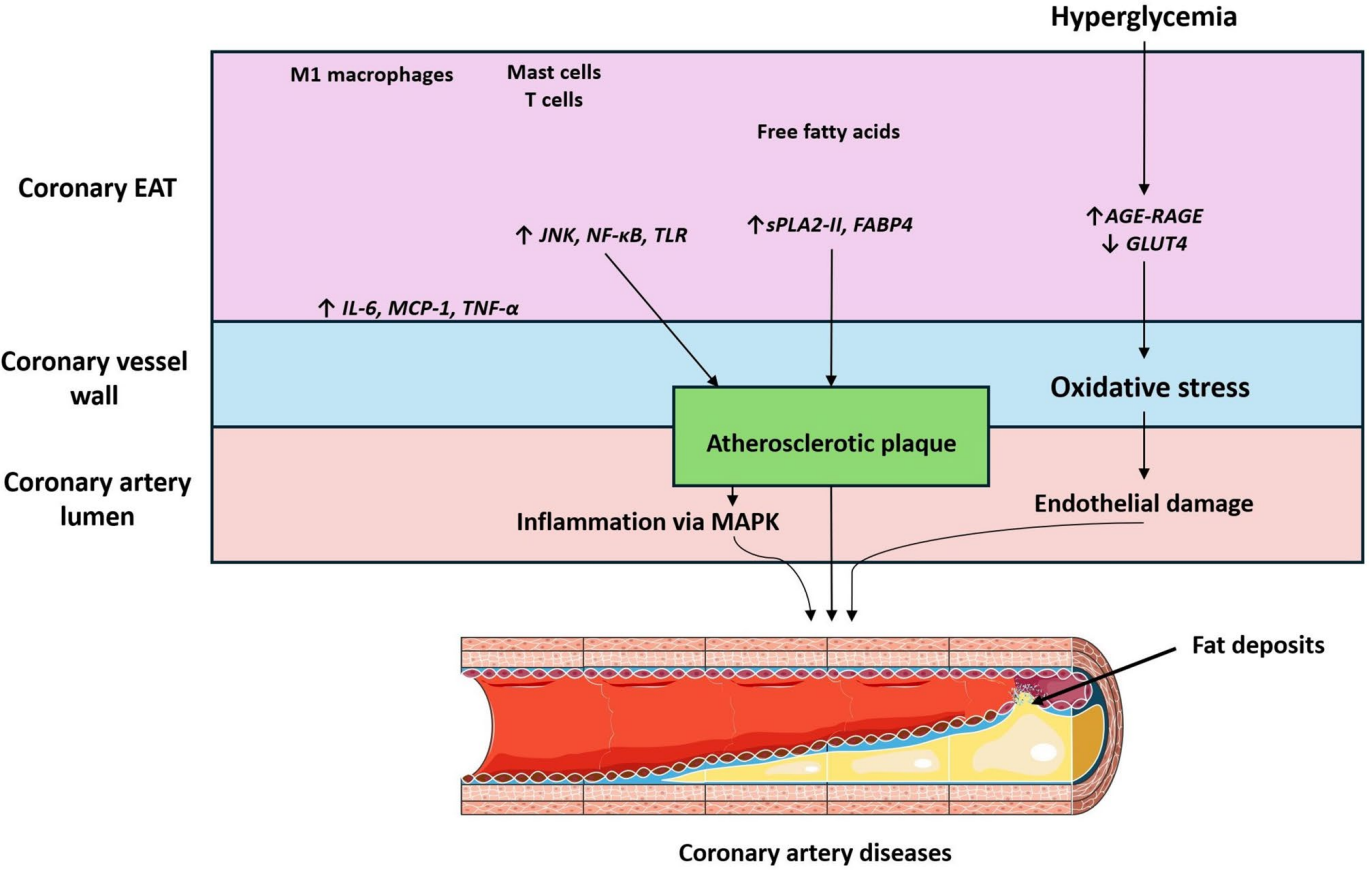
EAT post-MI: Following myocardial infraction or heart attack, the damaged heart tissue experiences hypoxia, triggering EAT to release extracellular vesicles containing angiogenic and prosurvival factors. These vesicles promote angiogenesis toward the infract site and influence gene expression in cardiomyocytes, leading to cardiac repair. There is an increase in cardiomyocyte markers and decreased fibroblast biomarkers during the healing phase, indicating successful tissue regeneration

EAT function and morphology alter during the pathological stress of metabolic disorders and diabetes. The protective responses are impaired as EAT promotes the development of CVD, owing to its anatomical and unobstructed contiguity to the coronary arteries and myocardium. Aside from vicinity, the quantity and activity of EAT play critical roles in cardiac function [11]. Besides pathological conditions, age, race, body mass index (BMI), and gender, are associated with anatomical alteration of EAT mass and distribution in the human heart, leading to structural remodeling and abnormal impulse generation. This impairs cardiac function because of heart failure, fibrosis, and neural dysregulation observed in CVD. By the age of 65 years, EAT thickness increases by almost 22% [48]. The thicker the layer of EAT, the greater the inflammatory activity via excess pro-inflammatory EAT-derived adipokine secretion, increasing the severeness and progression of coronary atherosclerosis. Notably, Caucasian women with higher BMI are characterized by greater epicardial fat thickness, with the lowest reported cases being black men with lower BMIs [48]. In patients with CAD, EAT thickness was related to the severity of CAD (determined by the Gensini score) [49]. Patients with an EAT thickness > 7 mm are associated with diastolic dysfunction [50]. Given the strong clinical correlation between EAT thickness and cardiovascular outcomes, regularly measuring EAT density is a valuable indicator for evaluating cardiovascular and metabolic risk [48]. Non-invasive imaging modalities, including echocardiography, ultrasound, computed tomography (CT), cardiac magnetic resonance (CMR), and multidetector computed tomography (MDCT), quantify EAT thickness (ranging from 1 to 25 mm) and supplement with a marker for visceral adiposity accumulation [51]. The role of EAT in cardiac pathology is shown in Fig. 3.

### EAT Biochemistry

The unique biochemical profile of EAT makes it a critical player in cardiovascular health. The proximity to the myocardium allows paracrine and vasocrine effects of EAT, where secreted factors from EAT directly influence myocardial function and coronary artery health. In obesity and metabolic syndrome, EAT expands and is more inflamed, secreting higher levels of pro-inflammatory adipokines and FFAs, which contribute to coronary artery disease (CAD), atrial fibrillation, and heart failure (R). Mast cells, B lymphocytes, T lymphocytes, and dendritic cells in EAT express Toll-like receptors (TLRs), activating them by extracellular ligands such as fatty acids. TLRs recognize endogenous products released by damaged cells from adipose tissue hypertrophy [52]. Activated TLRs upregulate inflammatory molecules in EAT via nuclear factor-κB (NF-κΒ) and JUN N-terminal kinase (JNK). Baker et al. demonstrated elevated activation of NF-κB and JNK pathways in EAT biopsies of people suffering from advanced CAD [53].

Additionally, the oxidative stress experienced by EAT, coupled with its production of growth factors including vascular endothelial growth factor (VEGF), suggests an impact on angiogenesis and vascular function. Cellular stress mediator, mitogen-activated protein kinase 5 (MAP3K5), is highly expressed by epicardial adipocytes, inducing cellular apoptosis and endothelial dysfunction [54]. The imbalance between ROS and inflammatory factors results in chronic inflammation, endothelial dysfunction, and consequently, coronary atherosclerosis. A seminal study on serum indexes suggests that EAT thickness correlates with VEGF expression and epicardial fat volume of patients [55].

### EAT Phenotype Switching

EAT phenotype switching refers to the dynamic ability of EAT to shift between different functional states, particularly between a “(WAT)-like” phenotype and a “(BAT)-like” phenotype. This plasticity plays a critical role in metabolic regulation and cardiovascular health. Recent data from morphological profiling, transcriptional profiling, and proteomic analysis consistently demonstrated that EAT exhibits more BrAT and BgAT characteristics than WAT. Histologically, epicardial fat possesses molecular features characteristic of those found in vitro in BgAT. Physiologically, epicardial fat produces heat and non-shivering thermogenesis in response to cold temperatures through UCP-1-mediated heat production, similar to BrAT [7]. In neonates, EAT has brown fat-like properties and functions, with limited physical flexibility and responsiveness to external factors. With aging, the responsiveness of epicardial adipocytes to environmental, metabolic, and hemodynamic influences increases, gradually shifting thermogenic EAT for energy storage [11]. The brown fat-like activity of EAT declines with advancing age as they undergo a brown-to-white transition [56]. As the primary function of BrAT is to thermoregulate, while WAT’s is to secrete pro-inflammatory cytokines to aggravate local and systemic inflammation, phenotype conversion is expected to drive CVD. The conversion is driven by the excessive release of IL-6 under chronic inflammation. Wang et al. used rabbit atherosclerosis models to exemplify how IL-6 activates the JAK-STAT3 signaling pathway. The findings revealed the reduced expression of UCP-1 (a marker of BrAT) and increased the expression of leptin (a marker of WAT), inducing the phenotype conversion of EAT from BAT to WAT in a dose-dependent manner [57].

Chronic ischemic conditions, particularly in advanced CVDs, further diminish the brown fat-like activity within EAT [58]. Patients with advanced CAD displayed a downregulation of genes associated with adipocyte browning and thermogenic activation in EAT, coupled with a reciprocal increase in the expression of genes encoding pro-inflammatory cytokines. However, it is possible to restore the brown phenotype (re-browning) or facilitate the conversion from WAT to BAT to improve glucose and lipid metabolism. This attenuates coronary atherosclerosis, conferring beneficial effects in individuals with prolonged ischemic conditions [59]. GLP-1 receptor on EAT, when activated, induces brown fat activity, reduces inflammation, and improves cardiac function by increasing epicardial fat transcription factor heme oxygenase 1 (HO-1) and PPAR-γ coactivator 1-alpha (PGC1α). These observations have shown benefits in modulating inflammation, mitochondrial activity, and, consequently, left ventricular function [60].

### EAT in Cardiac Arrest and Post-MI Conditioning

**Fig. 4:**
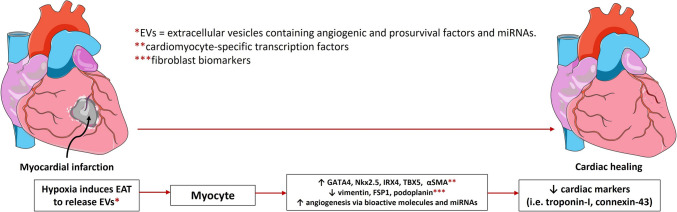
EAT and cardiac pathology: Epicardial adipose tissue (EAT) is implicated in the development of coronary artery diseases. Coronary EAT, containing M1 macrophages, mast cells, and T cells, releases free fatty acids under hyperglycemic conditions. A culmination of these factors induces oxidative stress, inflammation (via increased IL-6, MCP-1, TNF-α, JNK, NF-kB, TLR), and endothelial damage, leading to atherosclerotic plaque formation, ultimately narrowing the coronary artery lumen, and increasing the risk of CVD

The angiographic severity of acute coronary syndromes links increased EAT and the incidence of myocardial infarction (MI) [61] 62. Patients with coronary artery disease show a dense inflammatory infiltrate in their EAT, consisting of proinflammatory M1 macrophages, mast cells, and CD8+ T cells. These inflammatory cells release proinflammatory mediators, including IL-6 and TNF-α, into the coronary lumen contributing to systemic inflammation [63]. EAT inflammation contributes locally to coronary atherosclerotic plaque inflammation by inducing platelet aggregation and leukocyte activation followed by intravascular plugging, resulting in microvascular obstruction (MVO) and impaired coronary flow reserve [62]. Upregulation of the innate immune response signaling NF-κB, JNK, and TLR signaling, induces the secretion of inflammatory mediators from the coronary EAT [53]. Taken together, these pathological mechanisms result in a vicious circle of high EAT volume, inflammation, and arteriosclerosis, concluding in pronounced myocardial damage (Fig. 4).

EAT induces endothelial damage via oxidative stress. The excessive influx of FFAs is induced by the secretion of group II secretory phospholipase A2 (sPLA2-II) and adipocyte fatty acid-binding protein (FABP4) from epicardial adipocytes. FFAs infiltrate the adventitia, building up lipids in the atherosclerotic plaques of coronary arteries [3]. In people with diabetes, the co-occurrence of CAD and chronic hyperglycemia increases the damage to endothelial cells and oxidative stress. This process is driven by the binding of advanced glycation end products (AGE) to their receptor RAGE, reducing the levels of glucose transporter type 4 (GLUT4) [64]. CVD patients displayed epicardial adipocytes that overexpress cellular stress markers, such as kinases MAP2K3 and MAP3K5. These markers are linked to coronary inflammation and multiple proteases involved in lysosomal degradation and apoptosis [11].

The role of EAT in coronary atherosclerosis is used in clinical practice for early diagnosis and risk stratification. Patients with CAD exhibit greater EAT volume and thickness than individuals without atherosclerosis. Also, EAT volume is linked with the vulnerability of coronary artery plaques. Iacobellis et al. found dilated EAT volume in patients with non-calcified, vulnerable, unstable plaques compared to those with stable and calcified lesions. Coronary artery calcium (CAC) scores greater than 10 are associated with higher EAT volume, predicting the risk of atherosclerosis with a sensitivity and specificity of 72% and 70% [11] [65]. Notably, EAT contributes to developing early but not calcified coronary atherosclerotic plaques, which are highly unstable and vulnerable to rupture [11].

Notably, EAT has been linked to impaired reperfusion following percutaneous coronary intervention (PCI) [62]. Infract size predicts primary cardiovascular endpoints in patients after ST-elevation myocardial infarction (STEMI) [66]. Cardiac magnetic resonance imaging (CMR) is the gold standard for assessing infarct size, where EAT can be assessed [67]. Fisser et al. found that patients with acute MI showed a higher volume of EAT than healthy individuals, where increased EAT was linked to microvascular obstruction (MVO) and greater ST-deviation or infarct size following PCI [62].

### EAT in Cardiac Healing

Stem-cell transplantation has been extensively investigated as a promising therapeutic candidate for CVD [68]. Adipose tissue is currently utilized as a rich source of mesenchymal cells for regenerative therapy, showing promise in inducing spontaneous regeneration and cell therapy for MI-related tissue loss and cardiac remodeling. Adipose-derived stem cells (ASCs) have the capacity for ex vivo expansion and differentiation to other cell types, such as cardiomyocytes or endothelial cells. Importantly, ASCs release angiogenic factors that have immunomodulatory properties, making them ideal for ischemic tissue recovery. EAT-derived stem cells and their secretions have immense translational potential in cardiac healing owing to their cardiac specificity.

#### EAT-Derived Stem Cells (EATDS) in Cardiac Healing

Epicardial adipose tissue-derived stem cells (EATDS) exhibited a lower doubling time and higher cardiomyogenic potential compared to pericardial and omental ASC subtypes [69]. However, data from clinical studies are limited because the self-renewal capacity of stem cells is negatively influenced by the upregulation of cell death machinery. This results in decreased survival and differentiation signals in an ischemic and inflamed environment, a problem that affects the success of tissue regeneration [68]. Administering adipose-derived mesenchymal stromal cell secreted EVs were confirmed in embryonic rat cardiomyocytes exposed to hypoxic apoptosis, inducing the activation of the Wnt/β-catenin pathway, an effect counteracted by the Wnt/β-catenin inhibitor XAV939 [70]. Yamada et al. demonstrated an elevated ability of BAT-derived stem cells (BDSCs) to differentiate into cardiomyocytes (CMs) compared with cells derived from WAT [71]. The study showed that the replacement of newly developed CMs by BDSCs led to a reduction in the infarction area and an improvement in left ventricular function compared to WAT-derived stem cells or control. Notably, ASCs not only differentiate into CMs but also stimulate angiogenesis. Lambert et al. reported that human EATDS display a depot-specific angiogenic function, regulating the microvascular endothelial cell function by the release of microvesicles [69]. Angiogenesis is crucial for repairing injured or ischemic organs, and insufficient angiogenic activity in an ischemic heart limits revascularization, healing, and regeneration. While considering the influence of ischemia and inflammation in the cardiac tissue EATDS, the risk of further inflammation following transplantation offers translational challenges. However, preconditioning the tissues using pro-survival/healing cues addressed such challenges. Notably, Oses et al. demonstrated that subcutaneous human adipose tissue exposed to iron chelator deferoxamine, for diabetic neuropathic treatment, increased antioxidant capacity and expression of neuroprotective factors [72]. Additionally, immunomodulation using CRISPR/Cas9 gene editing holds promise in cellular preconditioning for EATDS transplantation. Meng et al., for example, treated diabetic mice with IL-10 to suppress the effected bone marrow environment, prior to MSC transplantation [73]. Similar approaches in EAT-specific, pro-angiogenic and anti-inflammatory factors to address the risk of recurrent inflammation are likely possible and warrant further research. Therefore, EATDS is a valuable subject for further studies for improved cardiac healing.

#### EAT/EATDS-Derived Secretions

EAT secretes different adipokines and extracellular vesicles (EVs), playing key roles in intercellular and intracardiac communication under physiological and pathological conditions. These secretions act in a paracrine, autocrine, and vasocrine manner, affecting nearby cardiac cells, coronary arteries, and the myocardium. Compared to other fat deposits, EAT has a higher number of adipocytes per gram despite their smaller size [74]. Also, the human EAT exhibits higher levels of saturated fatty acids and lower levels of unsaturated fatty acids compared to subcutaneous adipose tissue [75]. In patients with significant CAD, EAT exhibits significantly higher levels of chemokine (MCP-1) and several more inflammatory cytokines (IL-1β, IL-6, IL-6sR, and TNF-α) than subcutaneous fat. Accumulated FFA in EAT stimulates macrophages via TLR-4 activation inducing pro-inflammatory NF-κB to overexpress chemotactic cytokines (i.e., MCP-1, IL-6) [76]. The inflammatory mediators amplify vascular inflammation and plaque instability via apoptosis (TNF-α) and neovascularization (MCP-1) [77].

Furthermore, anti-inflammatory/-atherogenic adipokines released from EAT, such as adiponectin, were decreased under obesity, contributing to metabolic diseases and HF. Notably, omentin-1, a novel anti-inflammatory and insulin sensitizer adipokine derived from EAT, is reduced in patients with CAD [78]. Contrastingly, inflammatory reactions derived from EAT stimulate an angiogenic response and the development of collateral circulation in patients with obstructive CAD. Lately, the worsening of CAD has been significantly associated with a reduced adiponectin mRNA and an increased IL-6 mRNA level in EAT [79]. Moreover, EAT secretes molecules that promote the browning of white adipose tissue, shifting its phenotype toward a more metabolically active state, similar to BAT. This involves factors such as irisin, which enhances thermogenesis and improves glucose and lipid metabolism, potentially mitigating the development of coronary artery disease [80].

Besides chemokines and adipokines, EAT releases EVs that contain significantly different molecules depending on the stimulus. These molecules are categorized by different protein profiles and routes of formation into exosomes, microvesicles (MVs), and apoptotic bodies (ABs) [81–83]. In hypoxic conditions, EAT-EVs are enriched with angiogenic and pro-survival factors. The ischemic insults activate EATDS to secrete EVs packed with regenerative mediators to alter cardiac fibroblast (CF) gene expression toward CM lineage. Specifically, ischemia-challenged EVs released by EATDS upregulated CM-specific transcription factors, including GATA4, Nkx2.5, IRX4, and TBX5 in CF and the healing marker αSMA, while downregulating fibroblast biomarkers such as vimentin, FSP1, and podoplanin and cardiac biomarkers such as troponin-I and connexin-43 [84]. Lambert et al. found that EVs released by EATDS deliver miRNAs (i.e., miRNA126-3p) to target cells to promote microvascular endothelial cell migration and angiogenesis [69]. Cui et al. demonstrated the protective role of EVs isolated from rat EATDS in the myocardial viability of CM exposed to hypoxia/reoxygenation. Hence, the infusion of EATDS-EVs in a rat I/R reduces the myocardial infarction size and ischemia-induced myocardial enzymes such as creatine kinase–myocardial band (CK–MB), lactate dehydrogenase (LDH), and cardiac troponin I [85].

## Translational Potential and Future

Translationally, EAT is a measurable and modifiable cardiovascular risk factor; therefore, assessment of EAT via different imaging techniques needs to be readily accessible for clinical evaluations of CVDs. Dependence on EAT imaging alone could result in erroneous CVD prognosis. However, using cardiac MRIs, PET, and CT angiography in conjunction could alleviate non-specificity or incomplete risk assessment during screening. The biochemical profile of EAT can serve as a cardiovascular biomarker, with elevated volume linked to inflammation and atherosclerosis, detectable via CT or MRI for early risk assessment. Therapeutically, inducing EAT browning can enhance metabolism and reduce inflammation, while drugs such as TZDs and statins lower EAT volume and improve its profile. Novel non-invasive techniques are needed to monitor EAT browning in humans following a pathology and/or nutritional or therapeutical interventions. Lifestyle changes such as weight loss and exercise help reduce the adverse effects of EATs. Personalized treatments based on genetic and molecular signatures/characteristics offer promising, EAT-targeted interventions for high-risk patients.

The EAT mediates CVD through multiple pathways, including the excessive release of fatty acids and the activation of pro-inflammatory mediators that act on the myocardium and coronary vessels. These inflammatory signals from EAT could be attenuated with targeted agents such as GLP1R agonists and SGLT2 inhibitors, opening new venues for cardiovascular pharmacological interventions. Additionally, EAT and EATDS play critical roles in post-MI cardiac regeneration through the release of EVs enriched with angiogenic and prosurvival factors. EAT represents a depot of MSCs, possibly promoting cardiac regeneration following ischemic injury, particularly in response to ischemic insult, which is an aspect worth considering for cardiac management. EAT-derived EVs promote the proliferation of CM in experimental animals or human models of myocardial injury improving the contractile function.

The future of EAT research offers promising advancements in both understanding cardiovascular disease and developing innovative treatments. Precision targeting of EAT inflammation, focusing on pro-inflammatory secretions, including TNF-α, IL-6, and MCP-1, offers immense translational promise. Future therapies selectively modulating EAT-derived secretions to reduce cardiovascular damage without disrupting their normal functions are warranted. Interestingly, gene and cell therapies are emerging as promising interventions for EAT. Approaches including CRISPR/Cas9 could target genes involved in EAT inflammation and lipid metabolism, promoting browning and reducing adversity. Furthermore, EATDS-based regenerative strategies, creating a more favorable metabolic state, pose potent translational significance. To navigate the regenerative potential of EATDS, unexplored issues with the heterogeneity of the cell population, clinical scalability, and immunogenicity warrant further studies. Additionally, stem cell therapy-based challenges, including tumorigenesis, scar tissue, and undesired differentiation, require careful consideration for EATDS in translational cardiology. Notably, EAT has diagnostic potential for systemic diseases, as its properties reflect broader metabolic and inflammatory states.

Employing next-generation imaging techniques and AI-based algorithms is warranted to assess the critical role of EAT/EATDS in diseases such as type 2 diabetes, obesity, and metabolic syndrome, extending its relevance beyond cardiovascular health. Importantly, AI could help predict cardiovascular events by analyzing EAT volume, composition, and activity, allowing for more personalized treatment strategies and earlier interventions in high-risk patients. Moreover, AI techniques to address and detect long-term risks associated with the clinical application of EATDS offer immense translational potential in diagnosis and functional monitoring. Overall, the effective integration of the aforementioned data and comprehensive clinical indicators is imperative to maintain the accuracy of diagnosis and treatment using EAT-derived cells. In summary, the future EAT research spans diagnostic, therapeutic, and preventive realms and EAT-targeted interventions could revolutionize cardiovascular disease treatment, offering more personalized and effective healthcare solutions.

## Data Availability

Not applicable.
